# Transcriptomic insights into *Mycobacterium orygis* infection-associated pulmonary granulomas reveal multicellular immune networks and tuberculosis biomarkers in cattle

**DOI:** 10.1080/01652176.2025.2509503

**Published:** 2025-05-27

**Authors:** Rishi Kumar, Sripratyusha Gandham, Vinay Bhaskar, Manas Ranjan Praharaj, Hemanta Kumar Maity, Uttam Sarkar, Bappaditya Dey

**Affiliations:** a National Institute of Animal Biotechnology, Hyderabad, Telangana, India; b Regional Centre for Biotechnology, Faridabad, Haryana, India; c Department of Avian Sciences, West Bengal University of Animal and Fishery Sciences, Kolkata, West Bengal, India; d Department of Animal Genetics and Breeding, West Bengal University of Animal and Fishery Sciences, Kolkata, West Bengal, India

**Keywords:** Tuberculosis, bovine tuberculosis, *Mycobacterium orygis*, granuloma, transcriptome, biomarkers, zoonotic TB, RNA sequencing

## Abstract

*Mycobacterium orygis*, a member of the *Mycobacterium tuberculosis* complex (MTBC), has emerged as a significant contributor to tuberculosis (TB) in cattle, wildlife, and humans. However, understanding about its pathogenesis and severity is limited, compounded by the lack of reliable TB biomarkers in cattle. This study delves into the comparative pathology and transcriptomic landscape of pulmonary granulomas in cattle naturally infected with *M. orygis*, using high-throughput RNA sequencing. Histopathological analysis revealed extensive, multistage granulomatous, necrotic, and cavitary lesions, indicative of severe lung pathology induced by *M. orygis*. Transcriptomic profiling highlighted numerous differentially expressed genes and dysregulated pathways related to immune response modulation and extracellular matrix remodelling. Additionally, cell type enrichment analysis provided insights into the multicellularity of the granulomatous niche, emphasising complex cell-cell interactions within TB granulomas. Via comparative transcriptomics leveraging publicly available bovine and human TB omics datasets, 14 key immunomodulators (SOD2, IL1α/β, IL15, IL18, CCL2/MCP-1, CCL3/MIP-1α, CCL4/MIP-1β, CCL8/MCP-2, CCL20/MIP-3α, CXCL2/MIP-2, CXCL10/IP-10, CXCL11, and IFN-γ) were identified as potential biomarkers for active TB in cattle. These findings significantly advance our understanding of *M. orygis* pathogenesis in bovine TB and highlight potential targets for the development of diagnostic tools for managing and controlling the disease.

Bovine tuberculosis (bTB) is a persistent threat affecting both cattle and wildlife and serving as a potential source of zoonotic tuberculosis (TB) in humans globally. Although primarily associated with *Myco­bacterium bovis*, bTB can also be caused by other members of the *Mycobacterium tuberculosis* complex (MTBC), including the human tubercle bacillus- *M. tuberculosis* (Sawyer et al. 2023). Recent epidemiological studies have highlighted a surge in TB cases in cattle and wildlife attributed to *M. orygis*, an emerging member of the MTBC (Jawahar et al. 2024). A meta-analysis by Hugh et al. summarised the global incidence of *M. orygis* infection, reporting cases in 14 countries across five continents and affecting 17 host species (Hugh et al. 2025). Of these cases, 51% were in humans, while 49% occurred in livestock and wildlife, with cattle accounting for half of the animal infections (Basnyat et al. 2018). The highest incidence has been recorded in South Asia- particularly India, Pakistan, Nepal, and Bangladesh, which together account for 33.6% of global cases (Islam et al. 2023). Moreover, *M. orygis* has emerged as a primary cause of zoonotic TB in India, surpassing *M. bovis*, and indicating a potential shift in the epidemiology of zoonotic TB that presents new challenges to TB control efforts (Sumanth et al. 2023). Notably, a significant number of human cases have also been reported in low-burden TB countries such as Canada, Australia, and the USA, suggesting possible transmission linked to human migration (Lavender et al. 2013; Lombard et al. 2021; Riopel et al. 2024). The detection of *M. orygis* in non-endemic regions, along with its adaptation to multiple host species, raises concern about undetected ecological niches and the potential for cross-species transmission.

Despite the growing recognition of *M. orygis* as a leading cause of TB, our understanding of its pathogenesis and host immune responses, particularly in cattle, remains inadequate due to paucity of studies evaluating its pathogenesis, host immune response, gene expression profiling in either bovine or human host. Consequently, there is a need for a more thorough investigation into the molecular mechanisms underlying TB infection in cattle. Although, prior studies on various cellular models such as bovine peripheral encompassed blood mononuclear cell (PBMC), PBMC-derived macrophages, and bovine alveolar macrophages have deepened our understanding of bTB immunopathogenesis, no study until date has reported a comprehensive omics-based analysis of TB granuloma in naturally infected cattle (Rhodes et al. 2000; Hasankhani et al. 2022; Kumar R et al. 2023). In addition, except our recent study of early host responses to TB infection in the bovine using a bovine 3D pulmosphere model (Bhaskar et al. 2025), knowledge on the multicellular intricacies of pulmonary granuloma remained limited due to dearth of immuno-analytical tools for bovine cellular markers. Moreover, despite considerable research efforts, the identification of reliable biomarkers for confirming bovine TB remains challenging, hindering global disease management and control strategies (Borham et al. 2022).

Addressing this gap, our research endeavours to delineate the transcriptional landscape of pulmonary granuloma in *M. orygis*-infected cattle providing insights into the molecular mechanisms underlying host-pathogen interactions during *M. orygis* infection. *via* multimodal analysis of RNAseq data, we aim to- (i) characterise the multicellularity of active TB granuloma in cattle (Raudvere et al. 2019), (ii) identify the complex interactions between the core immune networks operative within pulmonary granulomas, and (iii) to determine potential host-immune biomarkers of active TB in cattle (McCaffrey et al. 2022). Furthermore, by leveraging comparative transcriptomic data from publicly available repositories encompassing bovine and human TB datasets, and juxtaposing our findings with existing knowledge, we aim to uncover commonalities in immune response pathways and molecular signatures across species. Such comparative analyses hold the promise of identifying conserved biomarkers with the potential to transcend species boundaries and serve as universal indicators of active TB infection.

## Materials and methods

### Sample collection

Lung tissues from cattle displaying suspected granulomatous lesions were collected during post-mortem inspections at an approved abattoir, under the supervision of a veterinarian., Granulomatous tissue samples were obtained from the right apical lobe of the lungs. Control samples were collected from the same lung lobes from a separate set of cattle with no evident pathological lesions in the lungs. Lung tissue samples were immediately washed with phosphate-buffered saline (PBS) and divided into three sections. One section was fixed in 10% buffered formalin (Sigma-Aldrich, USA), another was preserved in RNA stabilising and storage solution (RNAlater, Sigma-Aldrich, USA), and the third section was kept in PBS. Total DNA was extracted from the tissues preserved in PBS and subjected to PCR-based confirmation for the presence of *M. orygis* DNA. Extracted total DNA quality and quantity was determined by standard agarose gel electrophoresis and spectrophotometrically by measuring absorbance at 260 nm (Nanodrop 1000 instrument, Thermo Fisher, USA). The DNA were then subjected to PCR with *M. orygis-*specific primers (forward primer: 5′-gtggaaatggaagcgttgacc-3′; reverse primer: 5′-ggtacctcctcgatgaaccac-3′) as validated previously by Duffy et al. (2020) The PCR was performed using SapphireAmp^™^ Fast PCR Master Mix (Takara, Japan) in C1000 Touch thermal cycler instrument (Bio-Rad, USA). Following an initial denaturation at 94 °C for 1 min, 30 cycles of PCR involving a denaturation step at 98 °C for 5 s, an annealing step at 55 °C for 5 s, and an extension at 72 °C for 10 s per kilobase, concluding with a final extension at 72 °C for 5 min and an indefinite hold at 4 °C. In this study, lung tissues from crossbred Sahiwal x Holstein Friesian (SHF) cattle were evaluated (*n* = 3 each for healthy and diseased).

### Histopathology

Tissue samples were fixed in 10% neutral buffered formalin to preserve cellular architecture. Following fixation, samples were dehydrated through a graded series of ethanol (70%, 80%, 95%, and 100%), cleared in xylene (Merck, Germany), and embedded in paraffin wax (Merck, Germany). Paraffin-embedded tissues were sectioned at a thickness of 5 µm using a microtome and mounted onto glass slides. The sections were then deparaffinised in xylene (Merck, Germany), and rehydrated through descending grades of ethanol (Gemini Associates, India) to distilled water. Staining was performed by immersing the slides in haematoxylin (Merck, Germany), solution for 5–7 min to stain nuclei, followed by rinsing in tap water and differentiation in acid alcohol to remove excess stain. Furthermore, Eosin (Merck, Germany), staining was performed for 1–2 min to counterstain cytoplasmic and extracellular matrix components. After staining, the sections were dehydrated in ascending ethanol concentrations, cleared in xylene, and mounted with a permanent mounting medium (DPX) (Merck, Germany), Stained slides were examined under a bright-field light microscope (Carl Zeiss, Germany) to assess tissue morphology and architecture of the lung section (Slaoui and Fiette 2011).

### RNA extraction

RNA isolation was performed using a combination of TRIZOL (Sigma-Aldrich, USA) and the RNeasy Plus Mini - Kit (Qiagen, Germany). Briefly, approximately 100 mg of tissue was added to 1 ml of TRIZOL reagent and homogenised in a tube containing zirconia beads using a BeadBug microtube homogeniser (Benchmark Scientific, USA). Subsequently, 200 μL of chloroform (Sigma-Aldrich, USA) was added to the homogenate, which was then shaken for 15 s, incubated at room temperature for 3 min, and centrifuged at 12,000 g for 15 min at 4 °C. The upper aqueous layer was carefully separated for further RNA extraction using the RNeasy Plus Mini Kit, following the manufacturer’s instructions. Total RNA was eluted with 30 μL of RNase-free water and stored at −80 °C (Metzger 2024). The concentration and purity of the RNA were assessed using a Nanodrop 1000 (Thermo Fisher, USA), and the RNA-seq was outsourced to Nucleome Informatics Pvt. Ltd., Hyderabad, India.

### qRT-PCR

cDNA was synthesised from RNA using the Prime script first-strand cDNA synthesis kit (Takara, Japan) as per the manufacturer’s instructions and using a mixture of random hexamer and oligo dT primers. Primers were designed for bovine gene targets (SOD2, interleukin [IL]1α/β, IL15, IL18, CCL2/monocyte chemoattractant protein [MCP]-1, CCL3/macrophage inflammatory protein [MIP]-1α, CCL4/MIP-1β, CCL8/MCP-2, CCL20/MIP-3α, CXCL2/MIP-2, CXCL10/interferon γ-induced protein [IP]-10, CXCL11, and interferon [IFN]-γ) (Supplementary Table 1) using Primer-BLAST (NCBI) and real-time PCR was performed using a CFX96 Touch System (Bio-Rad, USA). Real time PCR protocol (GoTaq^®^ qPCR Master Mix, Promega, USA) started with an initial denaturation and enzyme activation at 95 °C for 2 min followed by 40 cycles of denaturation at 95 °C for 15 s, annealing and extension was carried out for 1 min at a temperature ranging from 55 °C to 65 °C (based on the target gene). Melt curve analysis was performed by heating the samples from 65 °C to 95 °C with an increment of 0.5 and fluorescence was recorded. Relative gene expression of the target genes was calculated using the 2^–ΔΔCT^ method with 60S Acidic Ribosomal Protein Large P0 (RPLP0) as an internal control (Schmittgen and Livak 2008).

### Whole transcriptome sequencing

#### RNA quantification, quality assessment, and library preparation

The RNA quality was assessed in a step wise manner, first by spectrophotometrically assessing the A260/A280 ratios and A260/A230 ratios followed by checking the RNA integrity *via* electrophoresis profile. All the RNA samples in this study attained the QC cut-off parameters required for RNA sequencing. The combination of precise quantification and integrity assessment ensured the selection of high-quality RNA suitable for downstream transcriptomic applications. For transcriptome library preparation, high-quality RNA samples were processed using the TruSeq^®^ Stranded Total RNA Library Prep Kit (Illumina, Cat# 15032618 and Cat# 20020596), which enables the depletion of ribosomal RNA and construction of strand-specific libraries. Post-library preparation, the final libraries were quantified using the Qubit^™^ 4.0 Fluorometer with the Qubit^™^ DNA High Sensitivity Assay Kit (Thermo Fisher Scientific, Cat# Q32851), ensuring consistent library input for sequencing. To assess the insert size distribution and overall library quality, the libraries were run on the Agilent TapeStation 4150 system using the High Sensitivity D1000 ScreenTape assay (Agilent Technologies, Cat# 5067-5582), in strict accordance with the manufacturer’s instructions. This step allowed for verification of successful library construction and detection of any unexpected adapter dimers or anomalies.

#### Sequencing data quality control and preprocessing

The quality of raw sequencing reads (FASTQ format) was evaluated using FastQC v0.11.9, employing default parameters to assess key metrics such as base quality scores, GC content, adapter contamination, and sequence duplication levels (Zhang and Kang 2021). Subsequently, raw reads were subjected to preprocessing using Fastp v0.20.1, a versatile and efficient tool for FASTQ file filtering and trimming. The following parameters were used during preprocessing: –trim_front1 9 –trim_front2 9 –length_required 50 –correction –trim_poly_g –qualified_quality_phred 30 (Chen et al. 2018). These settings were applied to remove low-quality bases, trim adapter sequences, correct mismatched base pairs, and eliminate poly-G tails typically introduced by Illumina platforms in low-diversity libraries.

After preprocessing, a second round of quality assessment was conducted using FastQC to ensure data cleanliness and integrity. All individual FastQC reports were then compiled and summarised using MultiQC v1.9, facilitating a comprehensive and comparative view of sequencing quality across all samples (Ewels et al. 2016).

#### Mapping of processed sequencing reads to the reference genome and analysis

The processed reads were aligned to the STAR-indexed *Bos taurus* ARS-UCD1.2 genome using the STAR aligner v 2.7.9a with specific parameters (‘–outSAMtype’ BAM SortedByCoordinate, ‘–outSAMunmapped’ Within, ‘–quantMode’ TranscriptomeSAM, ‘–outSAMattributes’ Standard) for *Bos Taurus* (ARS-UCD1.2) (Dobin et al. 2013). This alignment strategy was designed to efficiently handle high-throughput sequencing data while maintaining strand specificity and enabling downstream transcript-level quantification. To enhance the specificity of the alignment, rRNA features were excluded from the GTF file of the *Bos taurus* genome ARS-UCD1.2. This step was critical to prevent the non-informative alignment of reads to abundant rRNA regions, which could otherwise obscure genuine biological signal and skew gene quantification results. Subsequently, the resulting alignment files (sorted BAM) from individual samples were quantified using featureCounts v. 0.46.1, relying on the rRNA-filtered GTF file, to derive gene counts (Liao Y et al. 2014). This quantification process enabled the generation of a raw gene expression matrix representing transcriptomic profiles across all samples. The obtained gene counts were employed as inputs for DESeq2, and differentially expressed genes (DEGs) were identified after applying normalisation using the built-in default procedures in DESeq2. The sequencing depth for the RNA-seq data ranged from 200X to 400X, ensuring sufficient coverage for accurate gene expression analysis. The analysis was performed with specific parameters, including a threshold of statistical significance (–alpha 0.05) and the Benjamini-Hochberg method for p-value adjustment (Love et al. 2014). This approach controls the false discovery rate (FDR) and enhances the reliability of DEG identification. Functional enrichment analysis was conducted using ShinyGO 0.77 (Ge et al. 2020), and the results were cross-verified with g:Profiler (Raudvere et al. 2019) to ensure robustness. These analyses enabled the identification of biologically relevant pathways and gene ontology terms enriched among DEGs. Hierarchical clustering was applied to generate heatmaps, allowing the visualisation of expression patterns. This clustering approach enabled grouping of both genes and samples based on expression similarity, facilitating interpretation of expression dynamics. Discriminating variables between comparison groups were identified based on a stringent false discovery rate, with a threshold set at *p* < .05. These statistically significant differences formed the basis for further biological interpretation of group-specific transcriptomic alterations.

#### Cell type enrichment analysis

Cell type enrichment analysis is a crucial tool for discerning the prevalence of specific cell types within a set of genes. xCell, a sophisticated web tool, specialises in performing cell type enrichment analysis on gene expression data, focusing on 64 immune and stroma cell types. This powerful method is grounded in gene signatures derived from a wealth of knowledge acquired from thousands of pure cell types from diverse sources. xCell employs a cutting-edge technique designed to minimise associations between closely related cell types, enhancing the precision of its analysis. In the cell type enrichment analysis, we employed normalised read counts to ensure a consistent and unbiased assessment across samples. The use of raw scores in representing the outcomes ensures transparency and preserves the integrity of the original analysis results (Aran et al. 2017). The xCell tool provides 64 cell types, including lymphoid, myeloid, stromal cells, stem cells, and other cells. Hence, the xCell score analysis using the R package ‘xCell’ (https://github.com/dviraran/xCell) allowed us to obtain 64 immune cell type abundance scores. Web-based cell-type specific enrichment analysis (WebCSEA) available at https://bioinfo.uth.edu/webcsea/ is an application that provides a comprehensive exploration of the tissue cell type specificity of gene among human major TC map. This dataset comprises a total of 111 scRNA-seq panels of human tissues and 1355 TVs from 61 different general tissues across 11 human organ system. It provides a user-friendly interactive platform for a wide group of investigators to explore the cellular context of any gene list (Dai et al. 2022).

#### Network analysis

Differentially expressed genes identified as both up-regulated and down-regulated in the transcriptome were utilised to construct a Protein-Protein Interaction network using the STRING database (version 12.0) (von Mering et al. 2003). The full range of string network types, encompassing both physical and functional associations, was retained with a medium confidence level set at 0.4, following default settings. The generated network was then imported into Cytoscape software (version 3.9.1) for in-depth analysis of the PPI network structure and dynamics. During network analysis, highly connected clusters were identified using the molecular complex detection (MCODE) clustering method (Bader and Hogue 2003). This method helps uncover densely connected regions within the network. The cluster with the highest MCODE score in the network was selected for further analysis. Additionally, important genes or nodes were identified using six different topological analysis methods such as degree, closeness, radiality, betweenness, stress, and maximum neighbourhood component (MNC), were used to pinpoint individual nodes that play crucial roles in connecting different parts of the network or regulating network dynamics. Subsequently, the maximal clique centrality method was applied to identify the important hub genes based on the MCC Score (Chin et al. 2014). This comprehensive approach allowed for a thorough exploration of the PPI network structure and prioritise the selection of key functional modules and hub genes.

#### Comparative transcriptomics for validation of potential biomarker for bovine TB

We executed a comprehensive validation protocol to evaluate a set of genes identified as potential biomarkers for active TB in cattle. The validation process involved a meticulous comparison of these key genes with publicly available TB infection transcriptome datasets. Our specific emphasis was on TB disease cohort studies accessible through the NCBI Gene Expression Omnibus (GEO) database (https://www.ncbi.nlm.nih.gov/geo/). Detailed information, including the list of selected studies and their corresponding GEO accession numbers, can be found in Supplementary Data File S1. Our selection criteria were confined to two species: Bovine and Human, as detailed in our previous article. The dataset inclusion criteria encompassed studies involving *M. tuberculosis* and *M. bovis* infections specifically in PBMCs, whole blood, lung, and alveolar macrophages (AM). In broad terms, the analyses centred around comparing groups infected with *M. tuberculosis* and *M. bovis* with their respective healthy control groups. Based on the above inclusion factors 21 studies were considered for further comparison. The identification of DEGs was conducted using GEO2R, integrated with the limma package, with a focus on genes exhibiting statistically significant differences between pairwise groups (adjusted *p* value <.05, FDR <0.05). In instances where GEO2R was not accessible, Log_2_FC values were employed, provided they were available in the supplementary data of the respective articles.

## Statistical analysis

The statistical analysis for the study was conducted using a combination of bioinformatics tools and statistical software, and described under the respective sub-sections in the Materials and Methods section or in the figure legends. Especially, differential expression analysis was performed using DESeq2, where genes with a false discovery rate (FDR) of less than 0.05 and a log2 fold change greater than ±2 were considered significantly differentially expressed. A principal component analysis (PCA) was conducted to assess the variance and clustering of samples. Heatmaps and volcano plots were generated to visualise the expression patterns and the distribution of differentially expressed genes. The significance of the enriched GO terms and pathways was evaluated using a hypergeometric test, with a *p* value threshold of less than .05 considered statistically significant, and graphs are made using R (version: R 4.3.2). GraphPad Prism 9 was also used for data analysis and graph generation in selective cases as indicated in the corresponding figure legend. All statistical tests were carried out with a significance threshold of *p* < .05, corresponding to a 95% confidence interval.

## Results

### Severe disseminated granulomatous lung pathology in cattle infected with Mycobacterium orygis

**Figure 1.**
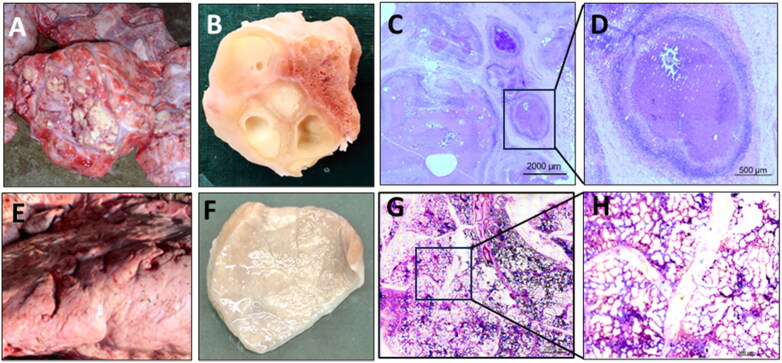
Pathological features of lungs of cattle infected with *Mycobacterium orygis*. Representative gross and histopathological images of (A–D) *M. orygis*-infected, and (E–H) healthy cattle lungs. Photographs of (A, E) cow lung tissues, (B, F) formalin-fixed tissue sections, (C, D & G, H) H&E-stained histopathological tissue sections from infected and healthy cattle, respectively. Gross and microscopy examination revealed typical granulomatous tissue morphology with the presence of caseous, necrotic, and cavitary lesions in the lungs of *M. orygis-infected* cattle compared to healthy cattle depicting intact lung parenchyma and alveolar structures. The bar depicts 2000 μm (C,G) and 500 μm (D,H).

Following confirmation of the presence of *M. orygis* DNA in the granulomatous lung tissue by PCR (Supplementary Figure S1)we evaluated the gross and histopathological characteristics of lung tissues (Figure 1(A–H)). Gross examination revealed the presence of numerous small to large tubercles dispersed throughout all lung lobes, indicative of an advanced stage of TB with disseminated granulomatous lung lesions (Figure 1(A)). Formalin fixed gross sections of lung revealed presence of various types of granulomas, including caseous, and cavitary lesions (Figure 1(B)). Histological analysis demonstrated the presence of well-organised classical granulomatous lesions representing various stages of granuloma formation, including caseous, liquefied, necrotic, and cavitary lesions, which are hallmark features of advanced TB disease (Figure 1(C,D)). Cavitary lesions of varying sizes, ranging from small size of a few millimetres to large size of several centimetres, were observed. The cellular composition within these granulomas was characterised by the presence of classical immune cells, including macrophages, lymphocytes, neutrophils, and multinucleated giant cells. In contrast, the gross and histopathology of the healthy cow lungs demonstrated normal lung tissue morphology and histology in case of healthy cows (Figure 1(E–H)). The observed pathologies in the case of *M. orygis* infected lungs underscore the intricate interplay between the pathogen and host immune responses, offering insights into the progression and pathogenesis of bovine TB lung granulomas associated with *M. orygis* infection.

### Significantly altered transcriptome profile in granulomatous lungs compared to healthy lungs

With our maiden approach to study the molecular immune responses underlying the severe granulomatous lung pathology observed in *M. orygis* infected cattle, we next performed whole transcriptome analysis of diseased lungs tissue and compared it with healthy cattle lungs. Post-processing of the RNA-Seq reads, alignment, and mapping to *Bos taurus* reference genome ARS-UCD1.2, normalised read counts were generated and the transcriptome data was deposited to the NCBI GEO database GEO273969. The transcriptome data quality parameters are provided in the Supplementary Data File S2. Subsequently, following the DESeq2 analysis, a stringent analytical framework was employed, wherein genes demonstrating at least a two-fold change, and with an adjusted *p* value of <.05 were deemed differentially expressed (DE). The transcriptome data analysis workflow was provided in Supplementary Figure S2.

**Figure 2.**
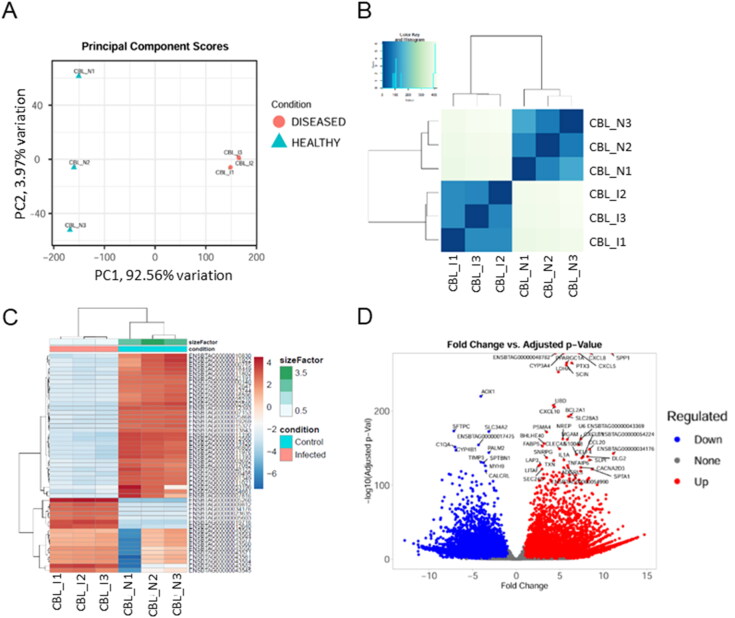
Comparative transcriptome analysis of *Mycobacterium orygis* infected and healthy cattle lungs. (A) PCA plot generated using DESeq2 data, showing variation within and between healthy and diseased groups. (B) Hierarchical clustering based on Euclidean distance using regularised log-transformed count data. (C) Heatmap of the top 50 differentially expressed genes, with colours indicating gene abundance from dark red (high) to light blue. (D) Volcano plot showing upregulated (red) and downregulated (blue) DEGs in *M. orygis*-infected versus healthy lung tissue, with FDR < 0.05 and Log2FC > 2. *N* = 3 per group, CBL_I: crossbred lung infected; CBL_N: crossbred lung normal; PC: principal component.

The PCA revealed distinct transcriptome cluster gene expressions in the healthy cow lungs and cow infected with *M. orygis*, as illustrated in Figure 2(A) (PCA).

The PCA plot represents the sample-to-sample distances within and between groups, emphasising the variation between these two groups. While the within group variation (PC2) was 3.97%, the between group variation (PC1) was 92.56%, indicating a significant clustering of the diseased samples from the healthy lung samples. Furthermore, as depicted in Figure 2(B), samples belonging to the same phylogenetic clades cluster closely, reinforcing the findings from the PCA analysis. Moreover, hierarchical clustering of the top 50 variable genes reinforces the clear separation between groups (Figure 2(C)). The hierarchical clustering analysis enhances our understanding of the molecular distinctions associated with *M. orygis* infection, highlighting the robustness of the observed separation between infected and healthy samples. A total of 27,607 variably expressed genes were plotted in the volcano plot depicting a significant number of DEGs in *M. orygis* infected cow lung granulomas relative to healthy cow lungs (Figure 2(D)). A total of 8349 DEGs (*p*adj < .05, >2log_2_ FC) were identified in the diseased lungs, of which 3016 DEGs were up-regulated, 5333 genes were down-regulated (Supplementary Data File S3). These DEGs were subsequently subjected to transcriptome signature-based granuloma cellular composition analysis and functional enrichment analysis.

### Inflammatory and immune-regulatory cell infiltration in the bovine pulmonary granulomas

**Figure 3.**
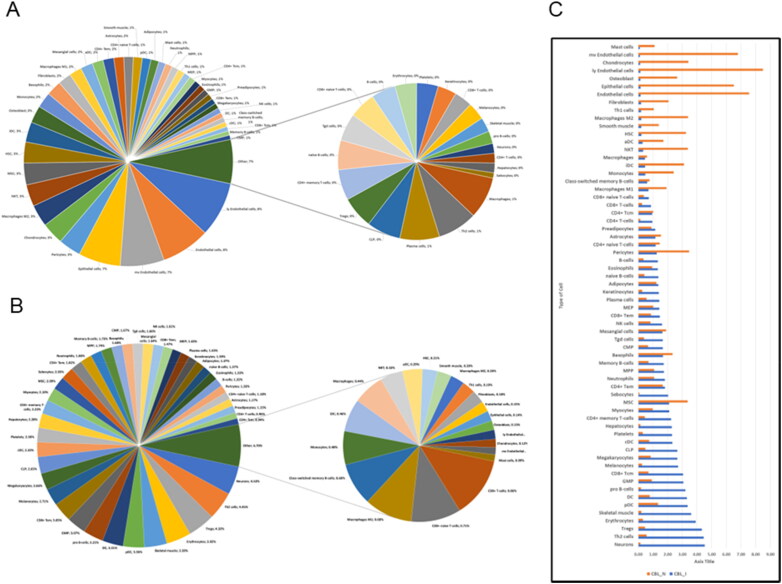
Alteration of the cellular composition of lungs following *Mycobacterium orygis* infection. Global cell type enrichment analysis was performed using xCell on normalised read counts. Pie charts display the proportions of various cell types in (A) healthy (*n* = 3), and (B) *M. orygis*-infected cattle lung tissues (*n* = 3). Colours in the pie charts are assigned randomly, representing cell proportions from highest to lowest. (C) Comparative analysis of cell type proportions between infected and healthy lungs.

Understanding the cellular composition of TB granulomas is critical as it provides insights into the disease mechanisms. We and progression indicating the phase of infection (Sholeye et al. 2022). Each cell type within the granuloma plays a specific role, influencing everything from disease control to progression towards active disease. Thus, dissecting the cellular architecture of TB granulomas is essential for advancing our understanding of the disease and enhancing our ability to combat it. For the cell type enrichment analysis, using xCell-web based tool (Aran et al. 2017), we used normalised read counts to ensure a consistent and unbiased assessment of global call-type analysis across samples (Figure 3(A,B)). This analysis identified 64 types of cells in both healthy and infected lung tissues however with considerable differences in their proportions underscoring the diverse cellular landscape present in the healthy and diseased lungs (Figure 3(A,B), and Supplementary Data File S4.1). A comparative analysis with the healthy lungs revealed a significant enrichment of multiple cell types within the *M. orygis* infected granulomatous lung tissues (Figure 3(C), and Supplementary Data File S4.2). Remarkably, several immune logically relevant cell types including, T-cells (Th2 cells, Tregs, CD4+ and CD8+ Tcm and Tem, and γδ T cells), B-cells (pro B-cells, memory B-cells, naïve B-cells, total B- cell, and plasma cells), CLP (common lymphoid progenitor cells), NK cells, and myeloid cells (DC, pDC, GMP, Megakaryocytes, Erythrocytes, Platelets, neutrophils, MPP, CMP and MEP) were found to be highly abundant in *M. orygis* infected granulomatous lungs compared to healthy lungs in cows. Notably, certain cell types unrelated to classical lung cells were also found to be enriched in the diseased tissue such as Neurons, Myocytes, Melanocyte, Hepatocytes, Sebocytes, and Keratinocytes (Figure 3(C)). Furthermore, using the DEGs of *M. orygis* infected cattle lungs over the healthy control, and a publicly available human TB lung granuloma transcriptome DEG data set, we performed a lung tissue-specific cell typing using the web-based tool WebCSEA (Supplementary Figure S3, and Supplementary Data File S4.3) (Love et al. 2014). While, the lung tissue-specific cell typing of bovine pulmonary granulomas identified 28 different cells types to be significantly enriched, human lung TB granuloma showed significant enrichment of 10 different cell types. Presence of highly significant number of a plethora of immune cells in the bovine lung granulomatous tissue indicate a highly active inflammatory state of the lungs highlighting active TB disease in the *M. orygis* naturally infected cattle.

### Functional enrichment analysis of differentially expressed gene

**Figure 4.**
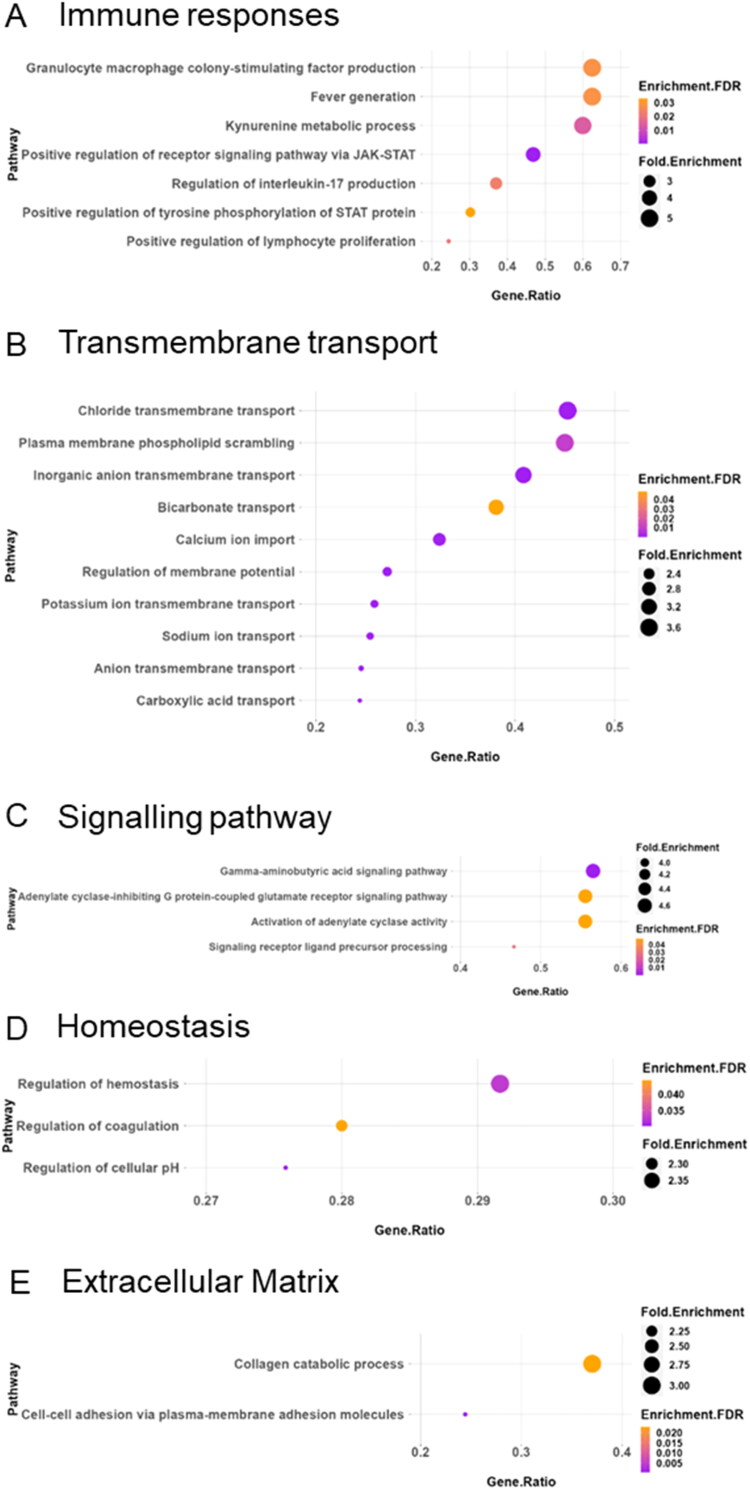
Pathway enrichment analysis of the up-regulated DEGs in *M. orygis* infected granulomatous lungs. Upregulated DEGs are enriched in the following biological pathways: (A) immune response, (B) transmembrane transport, (C) signalling pathway, (D) homeostasis, and (E) extracellular matrix. Analysis was performed using ShinyGO, and figures were prepared using R-Studio.

To gain insights into the biological implications of the observed differential gene expression in the case of granulomatous lung tissues compared to the healthy lungs, we performed Gene Ontology (GO) analyses on the upregulated and downregulated genes and proteins. Functional enrichment of the upregulated DEGs into the biological process reveals four major themes within the top GO terms: immune response, membrane transport, signalling pathway, and cellular homeostasis were up-regulated in the infected lung tissue compared with the healthy lungs (Figure 4(A–E)). Among the upregulated immune response related pathways, Granulocyte-Macrophage Colony-Stimulating Factor (GM-CSF) activity, kynurenine pathway, Receptor Signalling Pathways through JAK-STAT signalling, Interleukin-17 (IL-17) production, terpenoid metabolic process, and positive regulation of lymphocyte proliferation were the leading pathways (Figure 4(A)). In addition, significant up-regulation of several membrane transport pathways were observed including such as chloride, calcium, sodium, potassium, and mono- and dicarboxylic acid transport (Figure 4(B)). Moreover, the upregulated DEGs participate in diverse signalling pathways including GABA, cAMP and calcium signalling (Figure 4(C)), contribute to homeostatic regulatory biological processes within the immune system (Figure 4(D)), and Extracellular matrix remodelling (Figure 4(E)).

**Figure 5.**
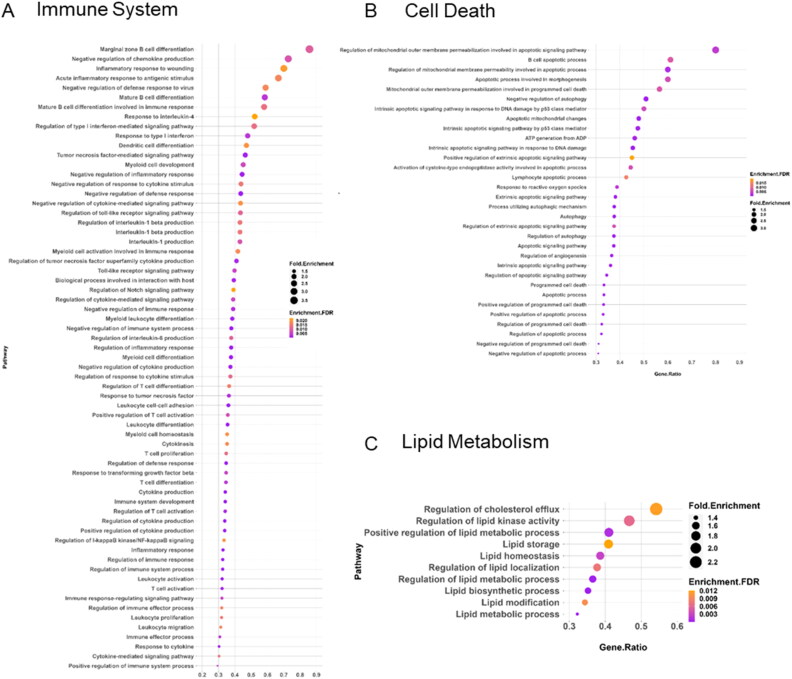
Pathway enrichment analysis of the down-regulated DEGs in *M. orygis* infected granulomatous lungs. Down-regulated DEGs are enriched in the following biological pathways: (A) Immune system, (B) cell death, and (C) lipid metabolism. Analysis was performed using ShinyGO, and figures were prepared using R-Studio.

Furthermore, down-regulated DEGs exhibit associations with various immune response pathways, including cytokine production, acute inflammatory response, and interleukin production, negative regulation of cytokines, chemokine production, defence response, leukocyte, myeloid cell, T cell activation, and differentiation (Figure 5(A)). Additionally, pathways related to cell death (Figure 5(B)), and lipid metabolism (Figure 5(C)) were also identified as top-downregulated pathways. For detailed information regarding the gene list associated with each pathway, please refer to Supplementary Data File S5.

### Protein-protein interaction network analysis of differentially expressed genes

**Figure 6.**
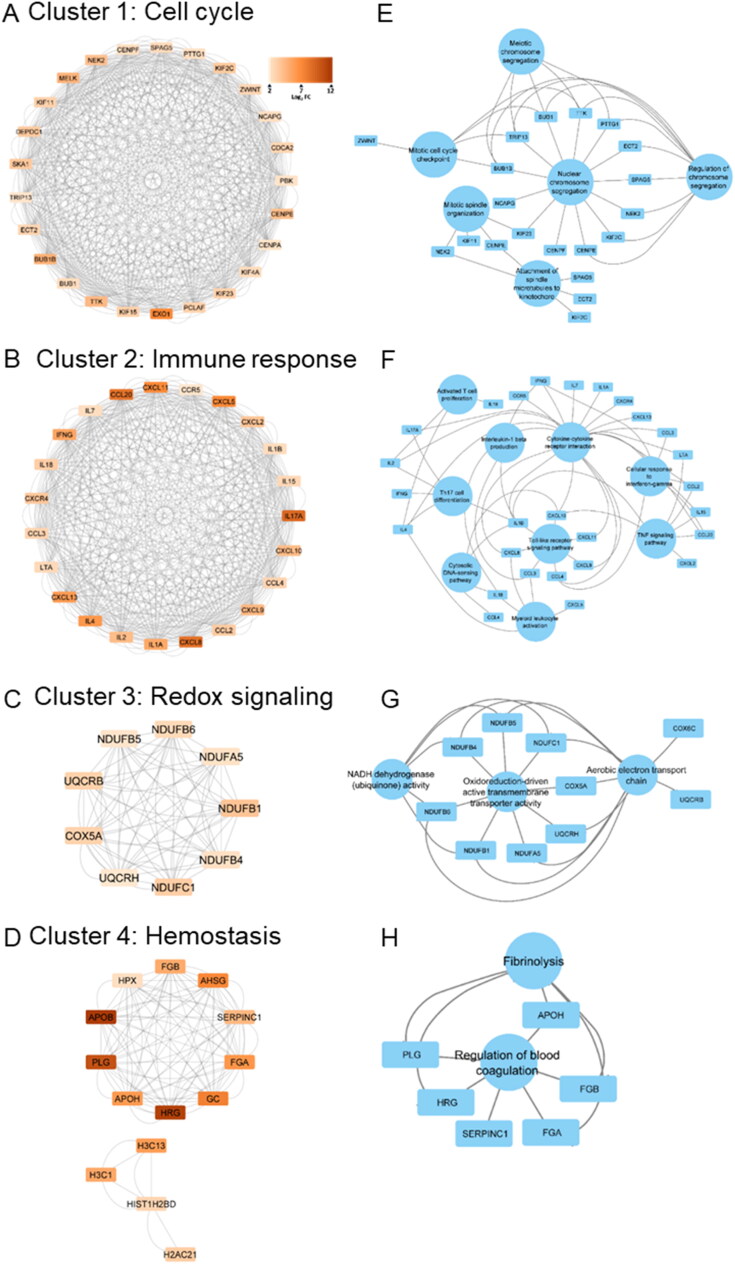
Critical gene network analysis of the up-regulated DEGs in the *M. orygis* infected cattle lungs: (A–D) Four key gene network clusters identified by MCODE in Cytoscape: (A) cluster 1: cell cycle, (B) cluster 2: immune response, (C) cluster 3: redox signalling, and (D) cluster 4: haemostasis. (E–H) Corresponding pathways for each gene network cluster were identified using the ClueGO plugin in Cytoscape.

To comprehensively explore the functional interactions among all up-regulated DEGs, we performed the construction of PPI maps utilising STRING software. Through this approach, we identified a potential network of interacting proteins. Subsequently, we subjected the significantly interacting proteins from the STRING analysis to further analysis using the MCODE algorithm within Cytoscape (Figure 6).

The top relevant MCODE clusters includes: cluster-1 (Cell Cycle), cluster-2 (Immune response), cluster-3 (Redox signalling), and cluster-4 (Haemostasis), respectively (Figure 6(A–D)). Furthermore, we ex­­panded the analysis to include the examination of associated pathways within each MCODE clusters using the CLueGO plug-in within Cytoscape. This comprehensive exploration encompassed pivotal pathways under cluster-1, including Meiotic chromosome segregation, Meiotic cell cycle checkpoint, nuclear chromosome segregation, regulation of chromosome segregation, mitotic spindle organisation, and Attachment of spindle microtubules to kinetochore (Figure 6(E)). Pathways such as Activated T cell proliferation, IL-1β production, cytokine-cytokine receptor interaction, Cellular response to IFN-γ, TH17 cell differentiation, Toll-like receptor signalling pathway, TNF signalling pathways, and Myeloid Leukocyte activation were identified under cluster-2 (Figure 6(F)). Under cluster-3, major pathways were related to oxidoreduction-driven active transmembrane transporter activity, NADH dehydrogenase (ubiquinone) activity, and aerobic electron transport chain (Figure 6(G)), while under cluster-4, fibrinolysis, and regulation of blood coagulation were two major pathways (Figure 6(H)).

### In silico identification and validation of potential biomarker for active tuberculosis

**Figure 7.**
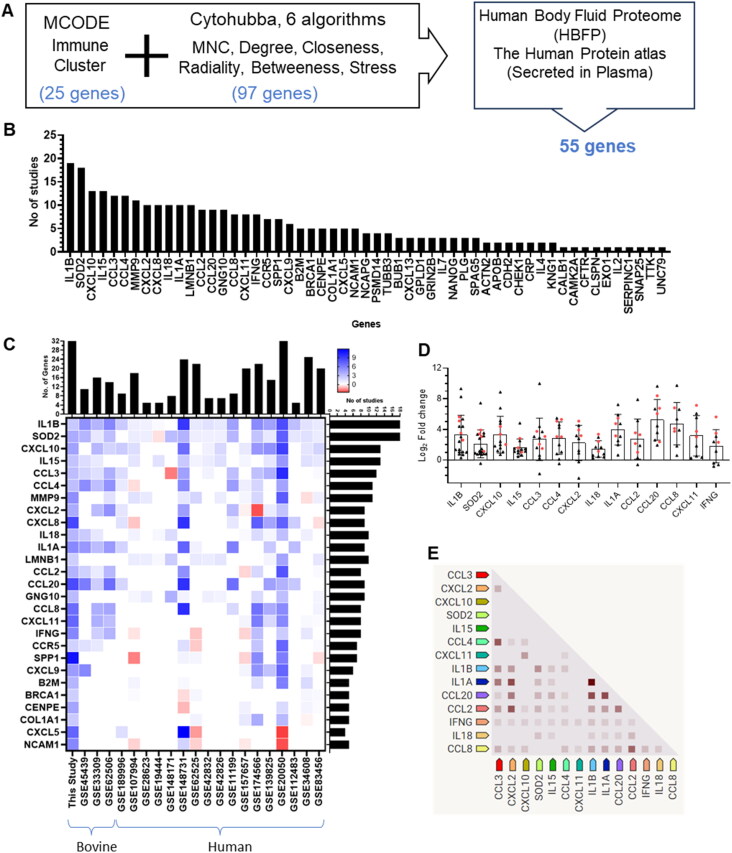
Potential biomarkers of active TB in cattle. (A) Identification of 122 genes *via* MCODE and CytoHubba analysis. (B) Detection frequency of 55 serum/plasma proteins across transcriptome studies. (C) Heatmap of Log2FC values for key genes detected in over five studies, with associated bar graphs showing the number of studies with gene upregulation and the number of genes detected per study. In the heatmap, shades of blue colour indicate upregulation, and red indicate downregulation. (D) Dot plot with mean ± SD representing Log2FC values for selected genes across 21 transcriptome studies. The black triangles indicate human studies, and the red dots indicate bovine studies. (E) Co-expression analysis of 14 shortlisted genes using STRING software.

From the total upregulated DEGs, a gene list was curated specifically targeting immune response-related genes associated with *M. tuberculosis* infection or TB diseases. This refined list aims to capture genes important for enhancing effective immune defence against TB, thereby providing additional insight into potential therapeutic targets and pathways for further investigation and intervention. In addition, Cytohubba analysis was conducted utilising six algorithms (MNC, Degree, Closeness, Radiality, Betweenness, Stress), revealing the top 50 PPI networks within each category (Supplementary Data File S6). Next, the selection of 25 genes from MCODE analysis, and 97 genes through Cytohubba analysis, resulted in a total of 122 unique genes after removal of duplicates (Figure 7(A)). Subsequently, additional analysis was conducted to assess the presence of these proteins in serum/plasma using the Human Body Fluid Proteome (HBFP) and the Human Protein Atlas database. Furthermore, each of these protein’s secretion status in plasma was thoroughly examined *via* extensive literature search, and 55 genes were selected for further analysis **(**Figure 7(B)).

For *in silico* validation, 21-selected publicly available datasets (including this study) on lung and PBMC transcriptomics were compared, of which 4 datasets were from bovine studies and 17 datasets were from human studies (Supplementary Data File S1). Of the 55 total genes, genes that were represented in less than 5 studies were excluded, and 27 genes were further shortlisted for checker board analysis. Figure 7(C) depicts a heat-map illustrating the expression pattern of these 27 genes associated with 21 transcriptomic studies along with their detection (presence or absence). Considering the selection of a bovine specific biomarker, first we shortlisted 14 genes (SOD2, IL1α/β, IL15, IL18, CCL2/MCP-1, CCL3/MIP-1α, CCL4/MIP-1β, CCL8/MCP-2, CCL20/MIP-3α, CXCL2/MIP-2, CXCL10/IP-10, CXCL11, and IFN-γ) that were upregulated in at least three of the four datasets from bovine studies including the current study. Interestingly, all these 14 genes were found to show upregulation in majority of the human transcriptome datasets highlighting the potential of the selected genes as transcriptional biomarker of active TB in the bovine and humans (Figure 7(D)). Furthermore, co-expression analysis of 14 genes using STRING confirmed that the selected genes are expressed together in different experimental conditions representing an optimised combination for developing multiple target-based biomarkers in active tuberculosis in the bovine (Figure 7(E)).

### qRT-PCR based validation of RNA-seq data

**Figure 8.**
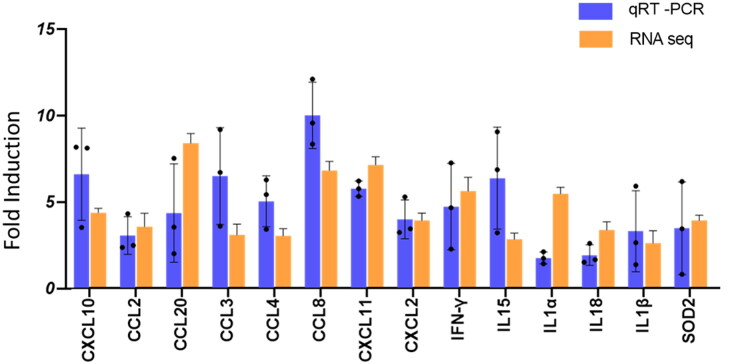
Relative mRNA expression of shortlisted genes by qRT-PCR. Real-time RT-PCR was conducted on 14 genes (SOD2, IL1α/β, IL15, IL18, CCL2/MCP-1, CCL3/MIP-1α, CCL4/MIP-1β, CCL8/MCP-2, CCL20/MIP-3α, CXCL2/MIP-2, CXCL10/IP-10, CXCL11, and IFN-γ). fold expression was calculated using the 2^−ΔΔCT^ method. Bars represent the average fold change values ± SD (*n* = 3) from qRT-PCR and RNA-seq analysis, comparing *M. orygis*-infected cattle lung tissues to healthy lungs. The dots in the qRT-PCR data represent individual samples.

To confirm the RNA-seq data, we performed qRT-PCR on all the 14 selected genes. Our qRT-PCR confirmed the significant upregulation of all the selected genes - SOD2, IL1α/β, IL15, IL18, CCL2/MCP-1, CCL3/MIP-1α, CCL4/MIP-1β, CCL8/MCP-2, CCL20/MIP-3α, CXCL2/MIP-2, CXCL10/IP-10, CXCL11, and IFN-γ which was in agreement with the RNA-seq data (Figure 8).

## Discussion

The study provides a comprehensive transcriptomic analysis of pulmonary granulomas in cattle infected with *M. orygis*, a less-studied member of the MTBC known for its zoonotic potential. Our findings not only enhance the understanding of *M. orygis* pathogenesis in the case of bovine pulmonary TB but also propose potential biomarkers for bovine TB, which could augment diagnostics and contribute to better disease management.

### Histopathological observation

Granulomas harbouring the tubercle bacilli serve as a distinctive niche where host immune defences intersect with bacterial survival strategies. Our study confirmed the presence of severe granulomatous, necrotic, and cavitary lesions in the lungs of cattle infected with *M. orygis*, which were indicative of an active but prolonged immune-mediated damage typical of TB (Hunter et al. 2023). These findings also highlight that the development and characteristics of TB granulomas due to *M. orygis* natural infection in cattle is similar to that reported in the case of *M. bovis* infection in cattle as well as *M. tuberculosis* infection in humans (Menin et al. 2013).

### Cellular composition

Our study provides unprecedented insights into the cellular composition of bovine TB granulomas, revealing a diverse array of cell types, including unexpected lung-unrelated cells. This comprehensive cellular landscape underscores the complex immune environment within granulomas and its role in disease progression. The identification of 64 distinct cell types, with significant differences in their proportions between healthy and infected lung tissues, highlights the intricate and dynamic nature of the granulomatous response to *M. orygis* infection. Remarkably, we observed an enrichment of various immunologically relevant cell types within the granulomatous tissues. These included T-cells (Th2 cells, Tregs, CD4+ and CD8+ Tcm and Tem, and γδ T cells), B-cells (pro B-cells, memory B-cells, naïve B-cells, total B-cells, and plasma cells), common lymphoid progenitor cells (CLP), NK cells, and diverse myeloid cells (DC, pDC, GMP, megakaryocytes, erythrocytes, platelets, neutrophils, MPP, CMP, and MEP). The presence of these cell types suggests a highly active inflammatory state and indicates the granuloma’s role in containing the infection and preventing its dissemination. Our findings are supported by previous studies that have emphasised the critical role of various immune cells in the formation and maintenance of granulomas and their importance in the immune response to TB (Pagán and Ramakrishnan 2014). The enrichment of these cell types within bovine granulomas aligns with existing literature and adds new dimensions to our understanding of the cellular dynamics in bovine TB. The comparative analysis with human TB lung granuloma transcriptome data, which identified a significantly smaller number of enriched cell types, further highlights the unique aspects of the bovine immune response to TB (McCaffrey et al. 2022). This comparison underscores the value of species-specific studies in understanding the pathogenesis of TB and developing targeted interventions.

Using transcriptomic data for cell type analysis is a new and powerful approach, providing an unbiased assessment of cellular composition. Tools like xCell and WebCSEA enabled detailed cellular typing, demonstrating the value of this method in understanding host-pathogen interactions within granulomas (Liang et al. 2021). Of particular interest is the identification of cell types unrelated to classical lung cells, such as neurons, myocytes, melanocytes, hepatocytes, sebocytes, and keratinocytes, within the granulomatous tissues. Previous studies have highlighted the role of non-immune cells in TB pathogenesis by modulating host immune responses and bacterial survival (Randall et al. 2015). A recent study by Monard et al. identified TUBB3^+^ axons and a novel TUBB3^+^ cell population with neuron-like morphology within TB granulomas in the lungs of mice, guinea pigs, primates, and human TB patients, suggesting the existence of a potential neuro-immune component within granulomas during TB (Monard et al. 2025). Randall et al. further demonstrated that *M. tuberculosis* can be readily phagocytosed by murine cultured neuronal cells, indicating that such cells may serve as a niche for bacterial persistence (Randall et al. 2014). In addition, airway smooth muscle cells have been reported to migrate to the lungs in response to chronic inflammation, contributing to airway remodelling (Gerthoffer 2008). Mesenchymal stem cells are also recruited to *M. tuberculosis*-infected lungs, where they may serve as niches for bacterial replication and dormancy (Fatima et al. 2020). Epithelial-mesenchymal transition (EMT) in *M. tuberculosis*-infected cells has also been shown to enhance inflammatory cytokine expression (Gupta et al. 2016). These cell types may express both epithelial and mesenchymal transcriptional signatures, a phenomenon that was evident in our study (Holgate et al. 2004; Kim et al. 2011; Qin et al. 2020). Notably, the migration of liver-resident Kupffer cells has been reported in acute respiratory distress syndrome (ARDS) and bacterial sepsis. In these conditions, acute liver injury compromises the organ’s filtration capacity, increasing the risk of lung damage (Herrero et al. 2020). In such scenarios, Kupffer cells migrate to the lungs and play a critical role in pulmonary protection by clearing altered platelets and coagulation products from the bloodstream. Given that chronic pathological changes in the liver during TB are well documented, the presence of Kupffer cells in the lungs during active TB may represent a protective mechanism against ongoing thrombotic and inflammatory insults (Dhiman et al. 2012). These findings suggest a more complex interaction between the immune system and other physiological systems than previously understood, potentially indicating systemic effects or migration of cells from other tissues in response to infection (Chandra et al. 2022).

### Transcriptomic findings

The results of our study demonstrate a complex interplay of molecular signalling in the pathogenesis of bovine TB, highlighting the significant upregulation of both signalling networks and PPI networks in granulomatous lung tissues. The functional enrichment of DEGs identified crucial pathways such as GM-CSF activity, JAK-STAT signalling, and IL-17 production. These pathways are instrumental in orchestrating a robust immune response, as evidenced by the activation and proliferation of lymphocytes, which are vital for the immune system’s ability to combat TB infection. Particularly noteworthy is the role of the JAK-STAT pathway, which has been extensively documented for its involvement in inflammatory and immune responses in various diseases including TB (O’Shea et al. 2015). The upregulation of this pathway suggests an enhanced activation state within the *M. orygis*-infected lung tissue, potentially facilitating the persistent inflammation characteristic of active TB granulomas (Boni et al. 2022). Furthermore, GM-CSF is known to play a pivotal role in the survival and function of tissue macrophages, which are key players in the pathogen survival and the host’s defence mechanism (Benmerzoug et al. 2018). Furthermore, IL-17, which is predominantly produced by Th17 cells, and has been implicated in promoting the formation and maintenance of granulomas *via* orchestrating the recruitment and activation of various immune cells to the site of infection (Torrado and Cooper 2010). Studies suggest that IL-17 enhances the recruitment of neutrophils and monocytes/macrophages to the granulomatous lesions, facilitating the encapsulation and isolation of the bacteria (Khader et al. 2009). While, this response is vital for the initial containment of the pathogen but can also contribute to exacerbated inflammation and pathology if not properly regulated, highlighting its dual role in TB pathogenesis.

Our analysis using STRING software and the MCODE algorithm in Cytoscape revealed significant clusters within the PPI networks that correspond to critical biological processes. Notably, the immune response cluster highlighted interactions that enhance cytokine production and T-cell activation, essential for an effective adaptive immune response against TB. This is supported by the identification of pathways such as Toll-like receptor and TNF signalling pathways, which are integral to initiating and sustaining the immune response in TB (Kaufmann and Dorhoi 2016). Moreover, the redox-mediated membrane transport and haemostasis clusters underline the metabolic shifts and vascular changes occurring in response to chronic infection (Pacl et al. 2018). These findings suggest that *M. orygis* infection in bovine lungs not only triggers a robust immune response but also induces significant metabolic and physiological adaptations that may influence disease outcome.

### Potential biomarkers

The identification of 14 key immuno-modulatory molecules (SOD2, IL1α/β, IL15, IL18, CCL2/MCP-1, CCL3/MIP-1α, CCL4/MIP-1β, CCL8/MCP-2, CCL20/MIP-3α, CXCL2/MIP-2, CXCL10/IP-10, CXCL11, and IFN-γ) as potential biomarkers is particularly noteworthy. These immuno-mediators are known to play pivotal roles in the recruitment and activation of various immune cells, reflecting the active immune surveillance and response in infected tissues (Zhuang et al. 2024). In the context of TB, SOD2, which is an antioxidant enzyme, may mitigate the oxidative damage caused by reactive oxygen species (ROS) produced during the immune response to *M. orygis* infection (Liao D et al. 2013). SOD2 was previously shown to differentiate between TB associated pleural effusions and malignant pleural effusion, suggesting its potential as a diagnostic biomarker for TB (Wang et al. 2014). Additionally, SOD1 was also proposed as a diagnostic marker for severe secondary pulmonary TB, along with S100A9, ORM2, and IL1F6 proteins (Liu et al. 2018). IL1α and IL1β are pro-inflammatory cytokines involved in the activation of macrophages and induction of other cytokines and chemokines, and are essential for the containment of *M. tuberculosis* infection and the formation of granulomas (Mayer-Barber et al. 2011). Prior studies in human TB patients showed enhanced levels of IL1α in serum, and IL1β in saliva (Diesch et al. 2021; Zhuang et al. 2024). Furthermore, given their roles in TB pathogenesis especially macrophage activation and IFN-γ production, both IL-15 and IL-18 have been investigated as potential biomarkers for TB (Wawrocki et al. 2019; Shaukat et al. 2023). Elevated levels of these cytokines in the serum have been associated with active TB disease, suggesting that they could be used to differentiate between active and latent TB infections. CCL2/MCP-1, CCL3/MIP-1α, CCL4/MIP-1β, CCL8/MCP-2, CCL20/MIP-3α, CXCL2/MIP-2, CXCL10/IP-10, and CXCL11 are chemokines critical for the recruitment of monocytes, macrophages, and lymphocytes to the site of *M. tuberculosis* infection (Yu et al. 2012; Domingo-Gonzalez et al. 2016). Particularly, Th1 cells are recruited by CXCL10, NK cells by CXCL11, and neutrophils by CXCL2 to the lungs (Rawat et al. 2013; Borkute et al. 2021). Furthermore, while CCL8 is involved in the recruitment of monocytes and T cells, CCL20 is known to be involved in the recruitment of dendritic cells and lymphocytes, contributing to the adaptive immune response (Rivero-Lezcano et al. 2010). In addition, CCL20 was highly expressed in the *M. tuberculosis* infected monocytes, and TB patients exhibited the up-regulated expression of CCL20, *via* MAPK/NF-κB-mediated transcriptional mechanisms (Rivero-Lezcano et al. 2010; Zhuang et al. 2024). CCL3, and CCL4 are known to be involved in the early stages of granuloma formation, while CCL2 plays a role in sustaining the granulomatous response and CCL4 was associated with disease severity (Hasan et al. 2009). Additionally, CCL4 was also detected in plasma and proposed as potential diagnostic biomarker, and with the combination of IP-10 (Sutherland et al. 2016). CXCL2 was identified as a potential biomarker for accurately diagnosing active TB from latent infection in outbred mice population and other lungs disease in human (Koyuncu et al. 2021). In addition, higher expression of CXCL2 was also reported in human TB patients, with reduction following treatment (Kumar NP et al. 2021). CXCL10 was reported to distinguish between different stages of TB infection, as well as drug-sensitive and drug-resistant TB cases (Sampath et al. 2023). Moreover, the CXCL10 release assay showed considerable sensitivity and specificity comparable to traditional IFN-γ release assay in TB patients with HIV co-infection and in immunosuppressed individuals (Pan et al. 2022). In case of bovine TB, several studies have not only reported heightened CXCL10 levels in both mRNA and protein levels in *M. bovis* infected cattle, but also proposed CXCL10 based bovine TB diagnostic platforms (Coad et al. 2019; Palmer et al. 2020). In addition, CCL4 was also reported as a potential diagnostic candidate for bovine TB (Khalid et al. 2022). CXCL11 plays a crucial role in TB by being a functional ligand of the CXCR3 receptor, contributing to macrophage and NK cell recruitment to infectious foci (Rawat et al. 2013; Torraca et al. 2015). Higher level of CXCL11 is reported in individuals with TB compared to healthy individuals indicating its diagnostic potential (Chung et al. 2015).

IFN-γ is a key cytokine in the immune response to TB, driving the activation of macrophages and the production of reactive nitrogen and oxygen species (Flynn et al. 1993). It is essential for the containment of *M. tuberculosis* within granulomas (Cooper et al. 1993). IFN-γ is the most sought-after cytokine, and was used in several formats for TB diagnosis in humans along with the standard ESAT-6/CFP-10 antigen specific IGRA test (Januarie et al. 2022). In a similar line, IGRA tests and other diagnostic platforms based on the same antigens as well as novel antigens have been in the development pipeline to be used for diagnosis of bovine TB replacing the age old tuberculin skin test (TST), which is considered as the gold standard (Vordermeier et al. 2016). As TST is known to provide false positive and false negative outcomes due to multiple reasons (Rodrigues et al. 2017; Didkowska et al. 2021), developing cell mediated immunity based diagnostic assays in a similar line to that of IGRA test wherein pathogen specific antigens can be used as a stimulating agent and supernatant can be screened for the fourteen biomarkers identified in this study to pin down a smaller set of biomolecules for TB detection in cattle that is more specific and sensitive. Such blood based biomarkers overcomes the inconsistency of nucleic acid based TB detection in cattle that could arise from unavailability of sputum from diseased cattle, complexity of nucleic acid extraction from milk or other body fluids, and technical limitation in collection of pre-scapular lymph node biopsies, etc (Goyani and Mukhopadhyaya 2023). Notably, in human the use of peripheral blood/serum as the diagnostic matrix circumvents the limitations associated with sputum-based diagnostics, particularly in pauci-bacillary cases, and is highly advantageous for populations where sputum collection is challenging or unreliable such as young children, and immunocompromised individuals (e.g. HIV-positive patients) (Kasule et al. 2024). Altogether, elevated levels of majority of these cytokines and chemokines have been associated with active TB in both humans and cattle (Domingo-Gonzalez et al. 2016; Khalid et al. 2022). Given their roles in immune signalling, these molecules hold promise not only for diagnosing bovine TB but also for monitoring disease progression and response to therapy in case of humans. This biomarker panel also supports cross-species diagnostic capability, aligning with the principles of the One Health framework, which emphasises integrated health solutions at the human-animal-environment interface.

### Limitation

While this study significantly advances our understanding of *M. orygis*-induced pulmonary granulomas, it is not without limitations. One of the limitations of this study is small sample size (*n* = 3) in each group, due to the availability of *M. orygis* infected lungs samples. Next, qRT-PCR validation was conducted using the same set of samples employed for bulk RNA sequencing rather than a larger cohort of clinical samples. As this limitation stems from the sample unavailability during the course of the project, we aim to extend this analysis as more positive cases become available in our future projects. Furthermore, as this study is based on total RNA isolated from granulomatous tissues involving a mixture of tissues involving various stages of granuloma development, the transcriptome does not address the complex nature of granuloma biology and the inter-granuloma variability of each stage of granuloma formation. This demands further investigation using tissue samples from different stages of granuloma formation as well as different phases of TB ­diseases. In addition, immunofluorescence-based characterisation of cell types should be included as an important parameter for future studies to further confirm and localise the identified cell population in the tissue sections. Finally, the potential systemic implications suggested by the presence of non-immune cells within granulomas warrant further exploration to fully understand their roles in TB pathogenesis (Alsayed and Gunosewoyo 2023).

### Conclusion

This study characterises the transcriptional landscape of pulmonary granulomas in naturally infected bovine lungs, providing insights into the pathogenesis of *M. orygis*. Histopathological assessment confirmed typical granulomatous TB pathology, while transcriptome-based cell type enrichment analysis revealed pronounced cellular heterogeneity within the granulomatous niche. Sixty-four distinct cell types were identified, with significant shifts in their abundance between healthy and infected lung tissues. Immune cell populations- including various T-cell subsets (Th2, Tregs, CD4^+^ and CD8^+^ Tcm/Tem, γδ T-cells), B-cells (naïve, memory, plasma), NK cells, and multiple myeloid lineages- were enriched in infected tissues. In addition, enrichment of non-­classical lung-associated cell types such as neurons, hepatocytes, and myocytes were observed, suggesting the possibility of systemic cell recruitment or broader host responses beyond the pulmonary immune compartment. Transcriptomic profiling of *M. orygis*-infected lungs revealed a broad, conserved immune signature consistent with those classically observed in *M. tuberculosis* and *M. bovis* infections. Comparative analysis across bovine and human TB datasets highlighted shared activation of key immunological pathways, including GM-CSF and JAK-STAT signalling, as well as GABA, cAMP, calcium signalling, lymphocyte proliferation, and matrix remodelling pathways. A 14-gene transcriptional signature associated with active TB was identified, demonstrating cross-species and cross-lineage relevance. Its consistency across hosts and *Mycobacterium* species, highlights its potential utility as a diagnostic biomarker for active TB in both veterinary and human health contexts. In summary, this study not only maps the transcriptomic complexity of *M. orygis*-induced granulomas but also identifies potential biomarkers and underscores the multicellular nature of TB inflammation. These findings offer a foundation for future efforts to develop improved diagnostics and interventions targeting bovine and zoonotic TB.

## Supplementary Material

Supplemental Material

## Data Availability

The transcriptome data are publicly accessible through the NCBI GEO accession number GSE273969.

## Software and database

Several publicly available databases were used to aid in the in-depth analysis of cell types, networks, and pathways based on the transcriptome data. Some of the major databases include NCBI-GEO, Human Body Fluid Proteome (HBFP) and the Human Protein Atlas database. All the databases and software information are provided in the Supplementary Data File S7.
