# Supplementary material for: Transcriptomic insights into Mycobacterium orygis infection-associated pulmonary granulomas reveal multicellular immune networks and tuberculosis biomarkers in cattle
Source: Vet Q. 2025 May 27;45(1):1–19. doi: 10.1080/01652176.2025.2509503 (PMC12120866; doi:10.1080/01652176.2025.2509503)
Supplement: Supplemental Material [file TVEQ_A_2509503_SM0774.zip › suppl_data/Supplementary_information.docx]

Supplementary information

**Title: Transcriptomic Insights into *Mycobacterium orygis* Infection-associated Pulmonary Granulomas reveal Multicellular Immune Networks and Tuberculosis Biomarkers in Cattle**

**Authors:**

Rishi Kumar^1,2^, Sripratyusha Gandham^1,2^, Vinay Bhaskar^1^, Manas Ranjan Praharaj^1,2^, Hemanta Kumar Maity^3^, Uttam Sarkar^3^, and Bappaditya Dey^1,2^*

**Affiliations:**

^1^National Institute of Animal Biotechnology, Hyderabad, Telangana, India

^2^Regional Centre for Biotechnology, Faridabad, Haryana, India

^3^West Bengal University of Animal and Fishery Sciences. Kolkata, West Bengal, India

***Corresponding Author:**

Dr. Bappaditya Dey Scientist-F

National Institute of Animal Biotechnology Hyderabad, Telangana, India - 500032 Telephone: 040-23120128

Email ID: [bdey@niab.org.in](mailto:bdey@niab.org.in) ORCID ID # 0000-0003-2728-4683

**Supplementary Figure S1**


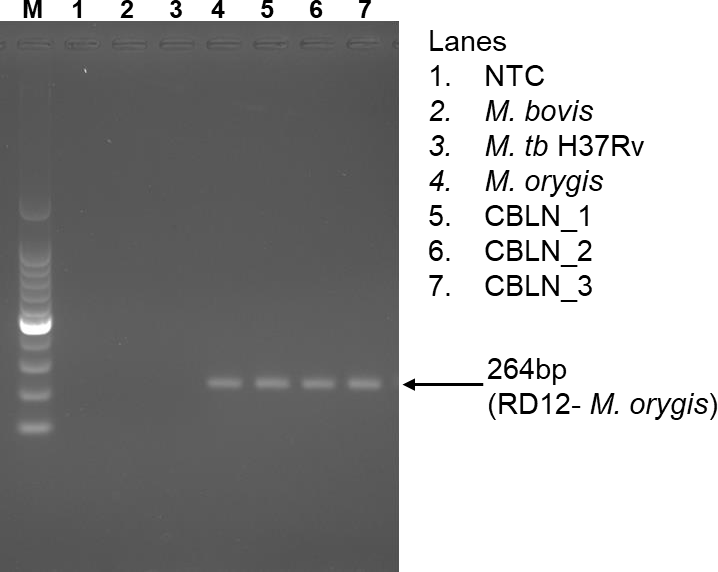


**Supplementary Figure S1. PCR based detection of *M. orygis* DNA in granulomatous lung tissue samples**.

**Supplementary Figure S2**


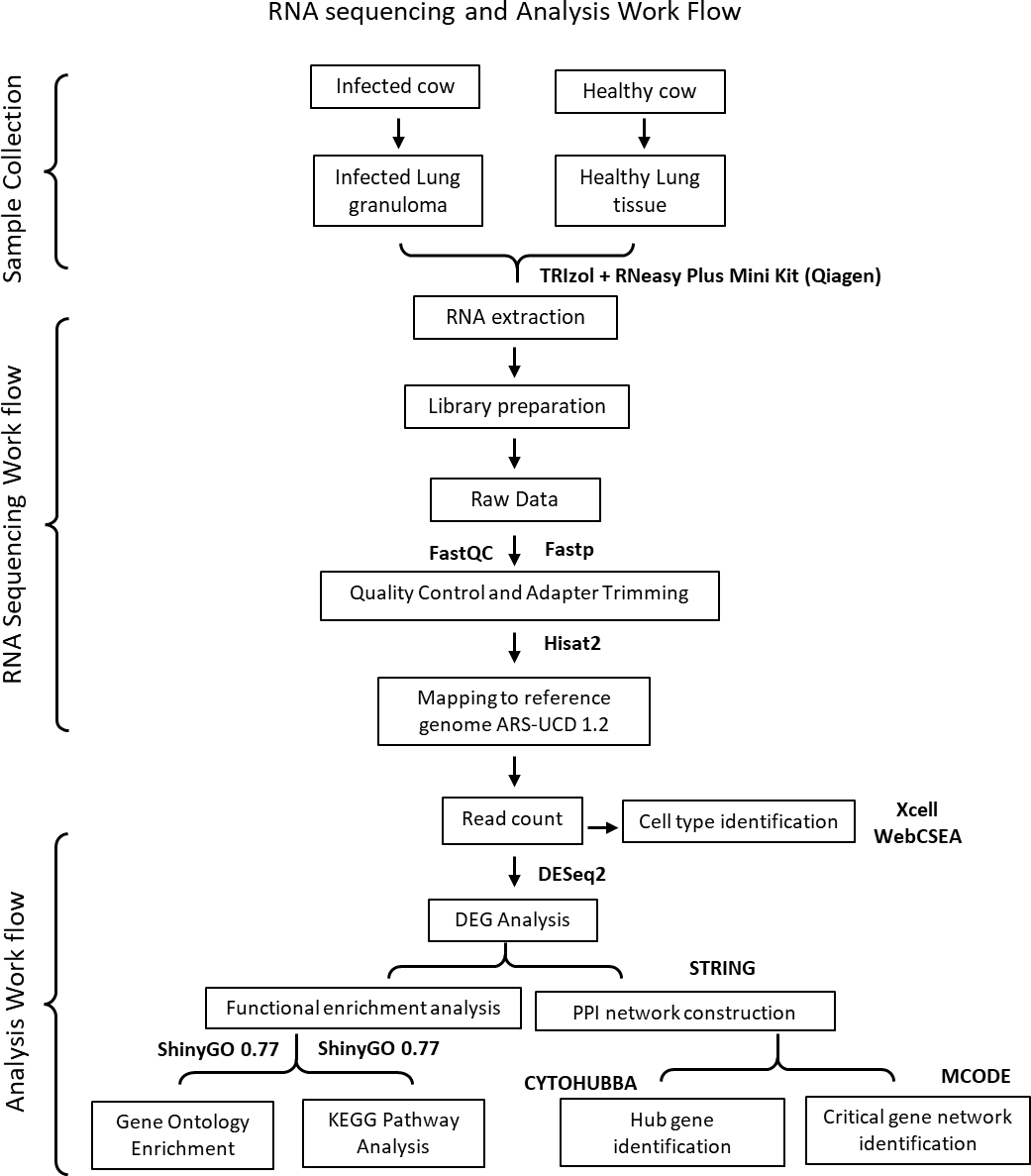


**Supplementary Figure S2. Transcriptome sequencing and analysis workflow.**

**Supplementary Figure S3**

Lung ciliated cell Respiratory goblet cell

Macrophage Basal cell Dendritic cell

Cd8-positive alpha-beta t cell Non-classical monocyte

NK cell Cd4-positive alpha-beta t cell

Type ii pneumocyte

B cell Plasma cell Club cell

Intermediate monocyte

**Cell Type**

Basophil Type i pneumocyte Vein endothelial cell

Lung microvascular endothelial cell

Alveolar fibroblast Bronchial vessel endothelial cell

Neutrophil Endothelial cell of lymphatic vessel Vascular associated smooth muscle cell

Classical monocyte Endothelial cell of artery Bronchial smooth muscle cell

Pericyte cell Adventitial cell Capillary aerocyte

Capillary endothelial cell

**Raw p-value**

**This Study Human Lung**

1.6600e-028 2.9500e-074

4.2400e-026 4.5800e-016

3.3400e-021 0.0023

9.4000e-019 5.5400e-013

9.4000e-019 0.9988

1.4300e-014 0.9063

2.8700e-014 0.7641

8.3300e-013 0.9892

1.2700e-010 0.9063

4.1500e-010 0.0001

7.4300e-010 0.8448

7.0600e-009 0.9997

1.2200e-008 2.2700e-017

2.0800e-008 0.6672

3.5400e-008 0.7641

5.9600e-008 1.5300e-038

1.8600e-006 0.2572

7.2000e-006 0.9988

0.0001 0.9748

0.0001 0.9997

0.0008 0.0791

0.0029 0.9960

0.0029 0.9063

0.0039 0.9063

0.0053 0.0489

0.0070 0.9892

0.0157 0.6672

0.0325 0.9960

0.0001

0.0005

0.001


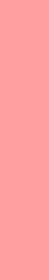


0.201

0.401

0.601

0.801

**Supplementary Figure S3. Differential cellular enrichment in the bovine and human tuberculous lungs tissues.** Lung tissue specific cell type enrichment analysis using WebcSEA on the DEGs yielded differential cell enrichment in the case of tuberculous lungs in cattle (this study) compared to the human lungs.

| **Table S1: List of qRT-PCR primers** | | |
| --- | --- | --- |
| **Primer Name** | **Nucleotide Sequence** | **Gene description** |
| *bIFN-γ | F, 5′-gctgattcaaattccggtgga-3′  R, 5′-caggcaggaggaccattacg-3′ | Interferon gamma |
| bIL-1β | F, 5’-cagtgcctacgcacatgtct-3′  R, 5′-ccagggatttttgctctctg-3′ | Interleukin 1 beta |
| bCCL-2/MCP-1 | F, 5′-tctcgctgcaacatgaaggt-3′  R, 5′- tgtatagcagcaggcgactt-3′ | Monocyte chemoattractant protein-1 |
| bCCL-20/MIP-3α | F, 5′- ctgcagcaagtcagaagcaagc-3′  5′- tgaagcccacaagaatactggg-3′ | Macrophage inflammatory protein 3 alpha |
| bCCL-3/ MIP-1α | F, 5′- cccacactccgtctcgca-3′  R, 5′- gccaaatggtgccgagaagac-3′ | Macrophage inflammatory protein 1 alpha |
| bCCL-8/MCP-2 | F, 5′- acaccgaagccttgaacctt-3′  R, 5′- tacacctggggaaggttggg-3′ | Monocyte chemoattractant protein-2 |
| bCXCL10/IP-10 | F, 5′- tcctcgaacacggaaagagg-3′  5′- gtccacggacaattagggct-3′ | Interferon γ-induced protein 10 |
| bCXCL11/I-TAC | F, 5′-gagtgtgaagggcatggcta-3′  R, 5′-ggcctatgcaaagacaccgt-3′ | Interferon-inducible T-cell alpha chemoattractant |
| bCXCL2/MIP-2 | F, 5′-accaaaccgaagtcatagcca -3′  R, 5′-cagccatccaagagcttctgt-3′ | Macrophage inflammatory protein 2 |
| bSOD2 | F, 5′-ggattgacgtgtgggagcat-3′  R, 5′-ggctgacggtttacttgctg-3′ | Superoxide dismutase 2 |
| bRPLP0 | F, 5′-cttcattgtgggagcagaca-3′  R, 5′-ggcaacagtttctccagagc-3′ | 60S acidic ribosomal protein large |
| bCCL4/MIP-1β | F, 5′-gcggaagattcctcgcaact-3′  R, 5′-atccacgtactcctggaccc-3′ | Macrophage inflammatory protein-1β |
| bIL1α | F, 5′-atcaagcccagatcagcaca-3′  R, 5′-gtagccgtcaggtatggacc-3′ | Interleukin 1 alpha |
| bIL15 | F, 5′-ccaaaacagaagcaaactggc-3′  R, 5′-agcactgcatcgctgttact-3′ | Interleukin 15 |

| bIL18 | F, 5′-aaccagggaaatcaacctgtct-3′  R, 5′-cagagatggttacggccaga-3′ | Interleukin 18 |
| --- | --- | --- |
| *b, Bovine. | | |
